# Supplementary material for: Transcriptomic Analysis of E. coli after Exposure to a Sublethal Concentration of Hydrogen Peroxide Revealed a Coordinated Up-Regulation of the Cysteine Biosynthesis Pathway
Source: Antioxidants (Basel). 2022 Mar 28;11(4):655. doi: 10.3390/antiox11040655 (PMC9026346; doi:10.3390/antiox11040655)
Supplement: Supplementary file 1 [file antioxidants-11-00655-s001.zip › antioxidants-1650692-Supplementary.pdf]

Supplementary Figures

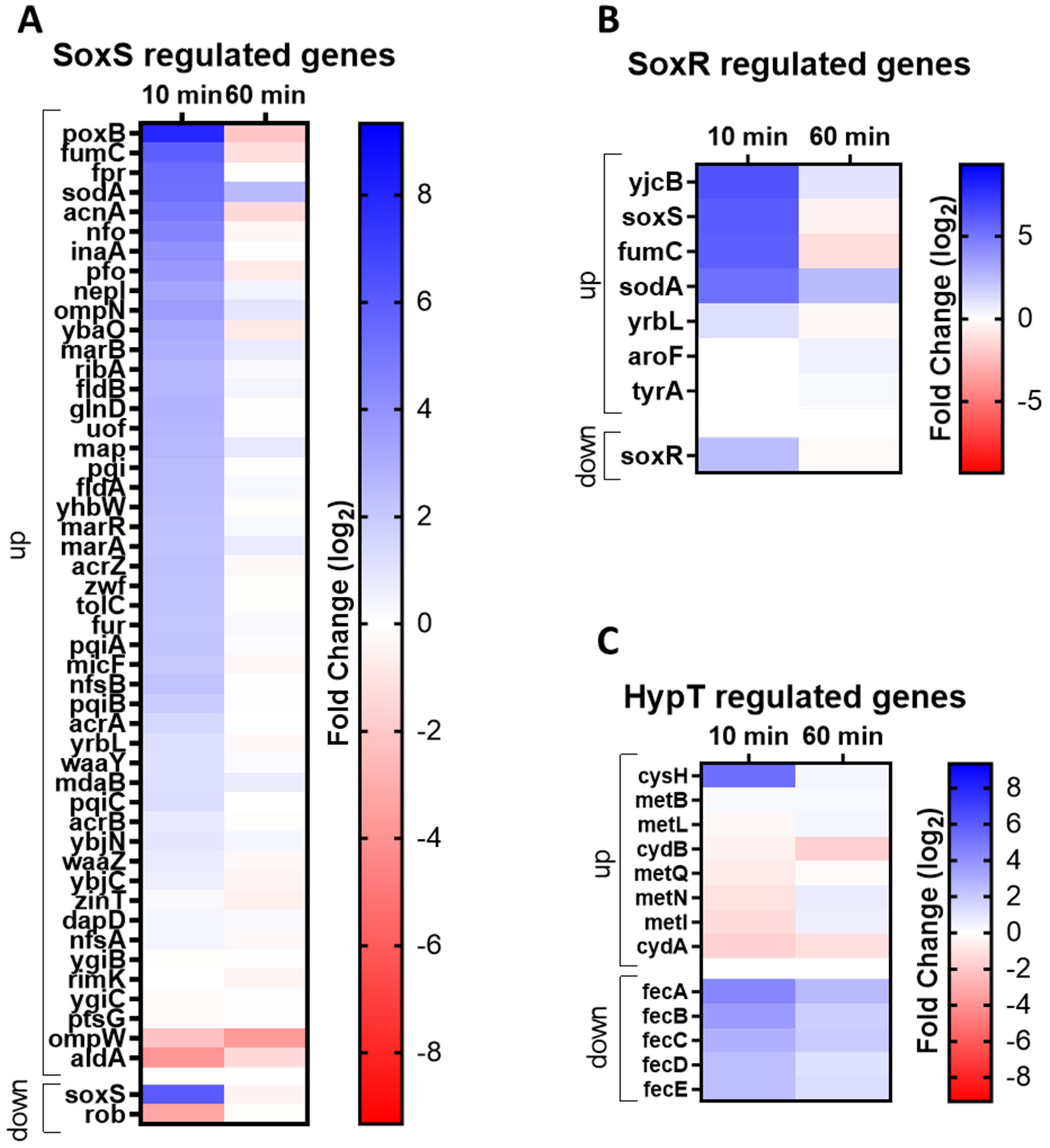

Figure S1. Heatmaps of genes regulated by the regulators SoxRS **(A, B)** and HypT **(C)** (involved in the response to oxidative stress). SoxRS were activated by H_2O_2, whereas the hypochlorite-sensitive transcription factor HypT did not display a pattern compatible with its activation. The color code corresponds to fold changes as compared to untreated cells at the respective time points (i.e. 10 and 60 min); n=4.

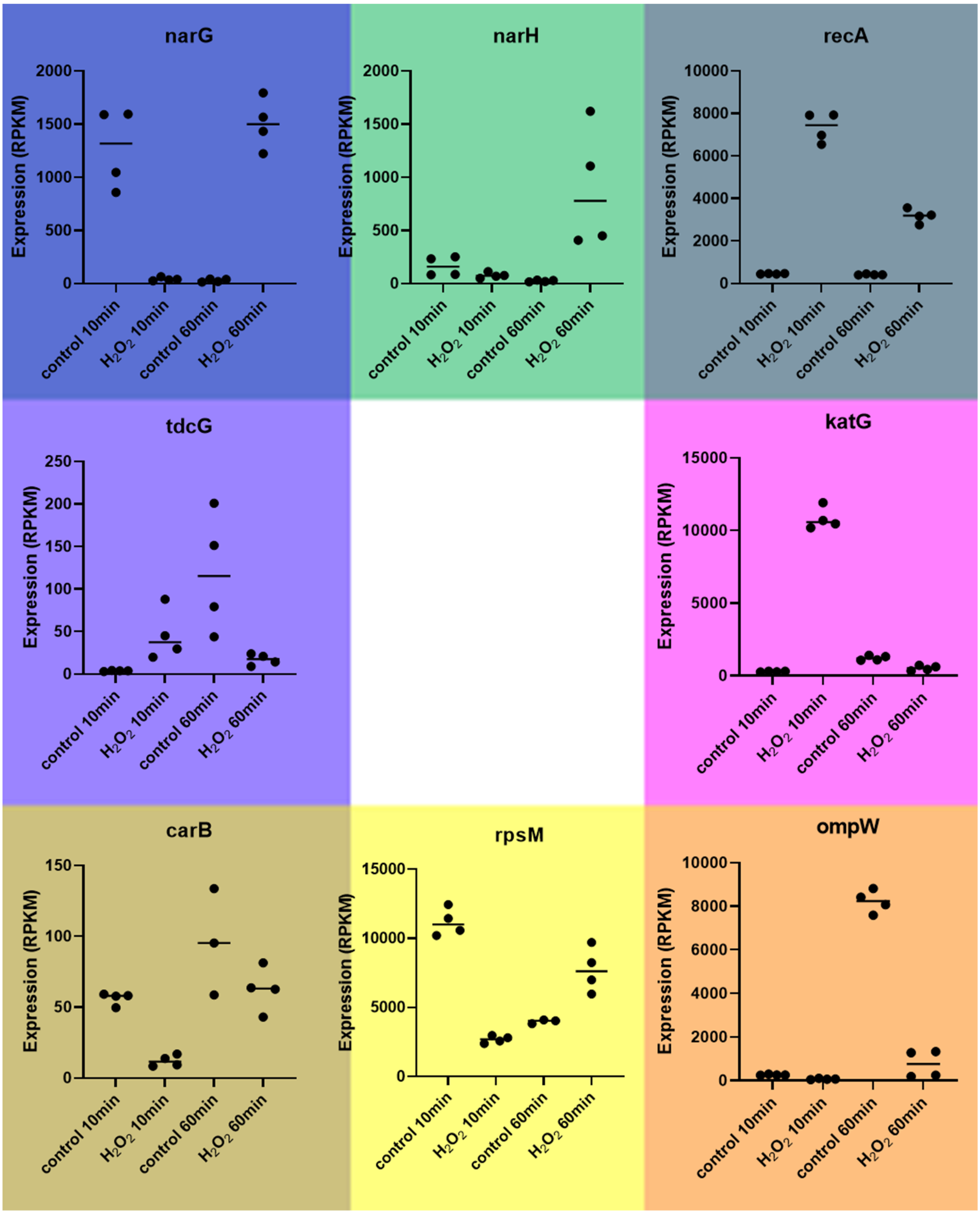

Figure S2. Expression levels of genes reresentative of the expression pattern represented in Figure 3; mean and individual values, n=4. Data are shown as reads per kilobase million (RPKM).

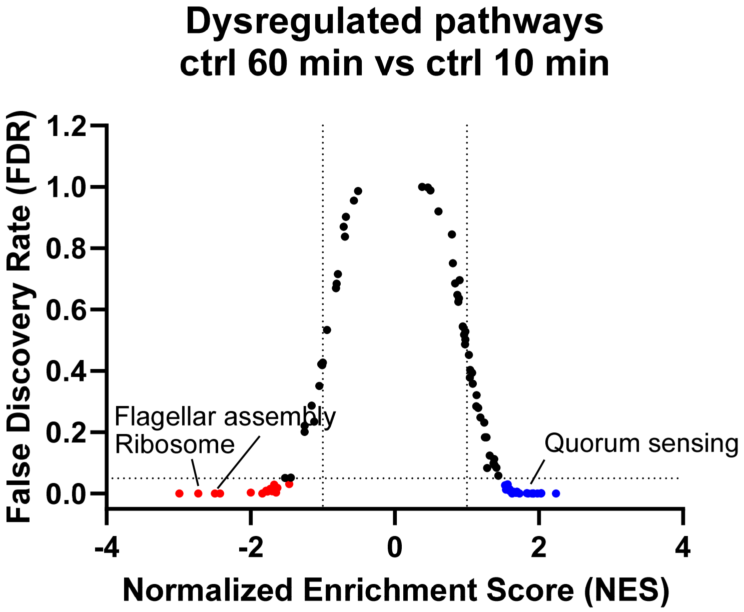

Figure S3. Dysregulated pathway comparing the untreated conditions at 60 min vs 10 min, using gene set enrichment analysis. Each dot represent a pathway from the KEGG database; thresholds: FDR=0.05, NES (normalized enrichment score) >1 and < -1.

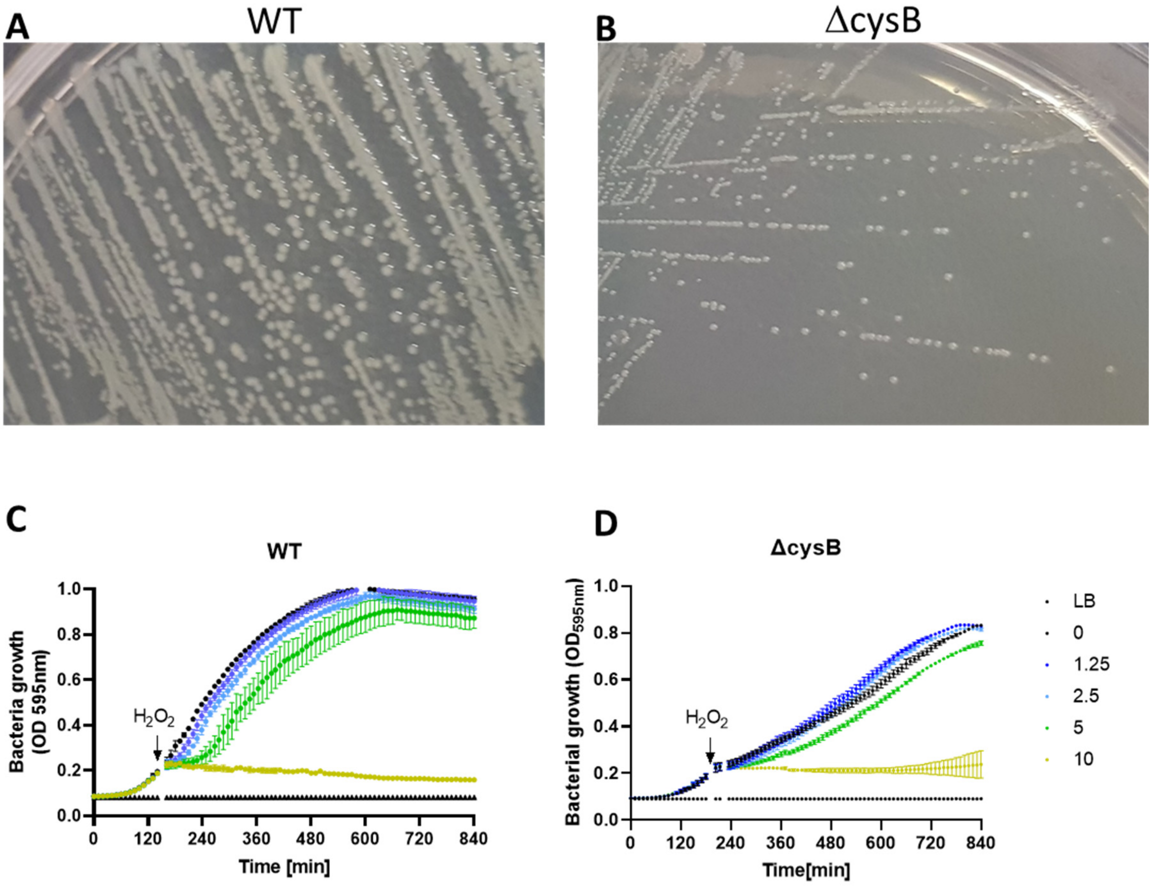

Figure S4. Impared fitness of *cysB* deleted mutant. **(A-B)** CysB deleted mutant displayed smaller and paler colonies on agar plate at the same magnification. **(C-D)** Growth in LB under increasing H_2O_2, added during the exponential phase (OD_{595nm}=0.2). *cysB* deleted mutant showed a slower growth but an identic sensitivity to H_2O_2. One representative experiment.
